# Engaging medical students in problem-based search and study of the biomedical literature

**DOI:** 10.5116/ijme.5975.c0bc

**Published:** 2017-08-17

**Authors:** Zhiyong Han, Samantha L. Margulies, Divya Kurian, Joshua M. Jabaut, Akshita Mehta, Ramzi Dudum, Huberta Koudoro, Ria S. Roberts, Jay Lee, Jonathan Li, Hieu T. Nguyen, Mark Elliott

**Affiliations:** 1 Department of Biochemistry and Molecular Medicine, The George Washington University School of Medicine and Health Sciences, Washington DC, USA; 2 Department of Obstetrics, Gynecology and Reproductive Sciences, Yale School of Medicine, New Haven, USA; 3 Department of Emergency Medicine, University of Pennsylvania School of Medicine, Philadelphia, USA; 4 Walter Reed National Military Medical Center, Bethesda, USA; 5 MedStar Harbor Hospital, Baltimore, USA; 6 The George Washington University School of Medicine and Health Sciences, Washington DC, USA

**Keywords:** Engaging medical students, problem-based search, biomedical literature

## Introduction

Studies indicate that many medical students have neither sufficient training to critically read and analyze biomedical research articles nor training in scientific writing and publishing.^1^^-^^5^ These two issues should concern medical educators given that, as physicians, medical school graduates will need to practice evidence-based medicine, which requires the practitioners to critically appraise the evidence of the validity and significance of published findings.^6^ Furthermore, writing and publishing scholarly articles are important skills for medical school graduates to have, especially for those who plan to become physician-scientists or academic physicians. Thus, a challenge for medical educators is how to effectively address these issues.^1^^-^^5^ In this perspective, we describe our approach of using patient-related problems, linked to biochemistry course materials students were currently learning, to teach students to search and study the biomedical literature to find specific answers and use their findings to write and publish review articles in professional journals. 

### Overview of the approach

In order to interest student to study the biomedical literature, faculty members (mentors) recognized that students need to see tangible purpose and value of doing it. Thus, mentors developed a list of overarching patient-related questions with uncertain answers but intimately linked to the biochemistry course materials their students were currently studying. The following is one such question. “Given that human skin is capable of synthesizing virtually all the vitamin D the body needs, why do many patients with gastrointestinal malabsorption disorders develop vitamin D deficiency?” These questions were very broad, ensuring that finding answers to these questions requires a serious effort to search and study the biomedical literature.

In order to incorporate into this process collaborative learning, which is effective for both individual learning that produces collective outcomes and collective learning that produces individual outcomes,^7^ students were asked to form small groups (2-4 students/group). Each group selected one question to answer under the guidance of a mentor. In order to not let the search and study of the literature interfere with students’ scheduled academic responsibilities, students were given significant autonomy to decide when and at what pace to search and study articles.

Then, the mentor taught students to develop and use proper query terms to find articles related to their questions in the PubMed database and to read and critically appraise the content of articles based on a set of established criteria and strategies.^8^ The mentor instructed students to search and study articles containing original studies, not Wikipedia or review articles that may influence their formative thinking. To help students not to become overwhelmed by trying to search for all the information at once to answer overarching questions, and to teach a student to think about an overarching question in multiple ways, the mentor helped students break down each overarching question into a series of smaller questions. Then, the students searched the PubMed database, identified relevant articles, read and appraised the findings in these articles, discussed their findings amongst themselves and with the mentors, organized their findings, and formulated answers to one smaller question at a time. Doing so ensured that students stay focused and make incremental progress in mastery learning one issue at a time.

The mentor also conducted parallel PubMed search to find articles, exchanged articles with students, and critically read all articles to acquire new knowledge and be able to provide clarification to students on their perception of an article’s content. The mentor acted as a Bloom’s tutor^9^ and worked around students’ schedules to have discussions with them as often as needed to help them assess what they knew and did not know, to address their missing prerequisite knowledge and to answer their questions concerning experimental design, methodologies, biostatistics, data interpretations, and conclusions in the articles they had studied or were reading. During discussions, the mentor asked students to clarify how they evaluated and appraised information before making any comments or suggestions, to trigger a sense-making cognitive process in the minds of students to formulate their own unbiased answers and conclusions. In short, the mentor’s task was to tutor students to achieve mastery learning.^9^

Students were required to use their findings to write narrative review articles. Specifically, the mentor asked students to study a published narrative review article as an example to learn the general structure of such an article and then use their findings to imitate the example article in writing a narrative review article of their own. During this process, the mentor acted as a “quality inspector” to review every student draft. The mentor promptly provided critiques, and suggestions for organization and integration of information and manuscript revision. Eventually, the mentor actively participated in manuscript writing to ensure that critical issues were addressed and the final product was scholarly, containing current information that would interest potential readers. After the students and mentor agreed on a final draft, the mentor selected a journal, explained his reasons for the selection to the students, and guided students to experience the publishing process. This included studying author guidelines, manuscript formatting, writing a cover letter to the journal editor, online submission of the manuscript to the journal, studying and responding to reviewers’ critique and editorial decisions, accepting rejection, revising the manuscript according to reviewers’ suggestions, and resubmitting revised manuscript to the journal, achieving successful publication.

### Outcomes and Lessons learned

Due to our effort, some students were able to publish in peer-reviewed professional journals. These student participants felt confident in their ability and skills to search the biomedical literature and to critically study and analyze findings in published articles. Additionally, they achieved in-depth learning of specific biochemistry topics in the context of medical relevance and patient care, which they otherwise would not have acquired from reading and studying of textbooks and articles as the class assignment or in journal clubs. Student participants especially appreciated the opportunity of learning scientific writing and gaining a deep understanding of the publishing process and recognized how challenging scientific writing and publishing are. However, our approach is time-consuming and labor intensive. Thus, some student participants terminated their participation shortly after they started because they could not find enough time to search and study a large number of articles without sacrificing a significant amount of the time that they needed to study regular course materials and participate in formally scheduled curricular activities, and furthermore, they felt that their effort would be “wasted” since there was no guarantee for a publication. Thus, we will need to find other ways to motivate these students. Additionally, our approach appears to be mostly applicable to training students who have time management skills to juggle disparate obligations and with characteristics of intrinsically motivated learners as described by Ryan and Deci.^10^ Therefore, we need to find alternative ways to train other students.

## Conclusions

We have developed an approach to engaging students in search and study of the biomedical research literature in the context of finding answers to broad, patient-related questions. Simultaneously, they achieve in-depth learning of the medical relevance of biochemistry topics. Additionally, this approach is useful for training students in scientific writing and publishing. We believe that our approach can be adapted and modified by other medical educators to train their students to develop a set of essential skills required for lifelong professional competency.

### Conflict of Interest

The authors declare that they have no conflict of interest.
